# Self-Compacting Recycled Concrete Using Biomass Bottom Ash

**DOI:** 10.3390/ma14206084

**Published:** 2021-10-14

**Authors:** Manuel Cabrera, M. J. Martinez-Echevarria, Mónica López-Alonso, Francisco Agrela, Julia Rosales

**Affiliations:** 1 Construction Engineering Area, University of Córdoba, 14071 Córdoba, Spain; jrosales@uco.es; 2 Civil Engineering School, University of Granada, 18010 Granada, Spain; mjmartinez@ugr.es (M.J.M.-E.); mlopeza@ugr.es (M.L.-A.)

**Keywords:** biomass bottom ash, self-compacting concrete, mechanical behaviour

## Abstract

In recent years, the use of self-compacting concrete has been a great advantage and garnered undoubted interest in construction. Due to the environmental impact caused by the consumption of natural aggregates in the manufacture of concrete, a more sustainable approach is needed. An approach for more sustainable construction is to use industrial waste such as bottom ash from the combustion of biomass as a replacement for natural aggregates. This research aims to use biomass bottom ash as a replacement for natural sand (10%, 20% and 30% replacement); in addition, by utilizing a crushing process of the bottom ash, the ash has been used as a filler replacement (replacement 20%, 40% and 60%). The fresh and hardened properties have been evaluated according to the standard. The results show the feasibility of using biomass bottom ash in self-compacting concrete, providing a sustainable alternative in order to minimise environmental impacts related to the extraction and depletion of natural resources.

## 1. Introduction

One of the most significant advances in the construction industry has been the development of self-compacting concrete (SCC), also called high-performance concrete by some authors. This type of concrete is known for its excellent deformability and resistance to segregation. It is a type of fluid concrete that does not need to be vibrated or compacted during the pouring process [1]. Many countries have used and adopted this method as a starting point to develop SCC [2,3,4].

Concrete, in general, has undergone great changes in its dosage to achieve better strengths and obtain better durability. To this end, many studies have included materials such as fly ash [5,6], fly ash with polymers [7], silica fume [8], metakaolin [9] additives, etc.

Although it has been developed with the aim of not needing vibration or compaction, SCC is a recent type of concrete that achieves greater resistance to compression and better durability compared to traditional concrete [10] mainly due to its fine particles, superplasticisers and viscosity modifiers [11,12].

More and more industries related to concrete manufacturing are trying to apply more efficient technologies by optimising existing resources. For a few years, industrial waste of different types has been used after an exhaustive study of its properties for the manufacture of more sustainable concrete for the environment.

Bignozzi and Sandrolini [13] demonstrated the properties of SSC made from recycled tire rubber. Ali and Al-Tersawy [14] used recycled glass to replace fine aggregate in proportions of 0%, 10%, 20%, 30%, 40% and 50%; they showed that recycled glass can be used successfully in SCC manufacturing. Ghernouti et al. [15] studied the properties of SCC that contained waste fibers from plastic bags.

Many studies with industrial waste focus on the use of recycled aggregates from construction and demolition waste. González-Taboada et al. [16] used recycled coarse aggregate as a replacement for natural coarse aggregate, Pereira-de-Oliveira et al. [17] studied the permeability of SCC made from recycled coarse aggregate and Kou and Poon [18] reported the fresh and hardened properties of SCC using coarse and fine recycled concrete aggregate.

However, other industrial wastes have not been studied much in the manufacture of SCC, as in the case of ash from biomass combustion. Currently, biomass combustion for electricity generation accounts for 45% of renewable energy in Spain, generating a volume of residue of 120,000 tons/year [19], of which 64% corresponds to fly ash, which is the finest fraction carried by combustion gases and is retained in the filters of the combustion chimney; at present, its main use is as a fertiliser in agriculture due to its high content of potassium [20,21]. On the other hand, 36% corresponds to biomass bottom ash (BBA), and its recovery is currently difficult. There are some studies of the possibility of using BBA as a construction material; for example, it has been incorporated as a substitute for sand or cement [22,23,24,25]. The incorporation of BBA has been studied as an aggregate in granular materials treated with cement and in the stabilisation of expansive soils for road sub-bases [26,27], and some authors reported the use of BBA in conventional concrete [28,29]. However, the use of BBA in self-compacting concrete has not been studied much.

In this context, this research aims to study the mechanical behaviour of self-compacting recycled concrete that incorporates BBA in the replacement of natural sand and crushed BBA as a replacement for natural filler and to determine the effect of its incorporation on its properties in fresh and hardened states. The results obtained can be very significant, both from the point of view of science, which currently does not have much literature, as well as for its practical use due to the reduction in waste, reduction in consumption of natural aggregates and environmental benefits in its application in this type of recycled concrete.

## 2. Materials

The materials that have been used for this type of concrete are those commonly used by companies that produce SCC.

### 2.1. Cement

Ordinary Portland Cement (OPC)(Cementos Portland Valderrivas S.A, Alcalá de Guadaíra, Sevilla, Spain) type I with medium-high resistance 42.5 MPa at 28 days with high initial resistance R was used (CEM I 42.5-R) according to ASTM C150. Its high initial resistance performance renders it suitable for the manufacture of SCC. As a cement belonging to the CEM I type, it is ideal for the manufacture of precast and prestressed, obtaining high resistance values. The composition is shown in Table 1.

### 2.2. Limestone Filler (F)

**Figure 1:**
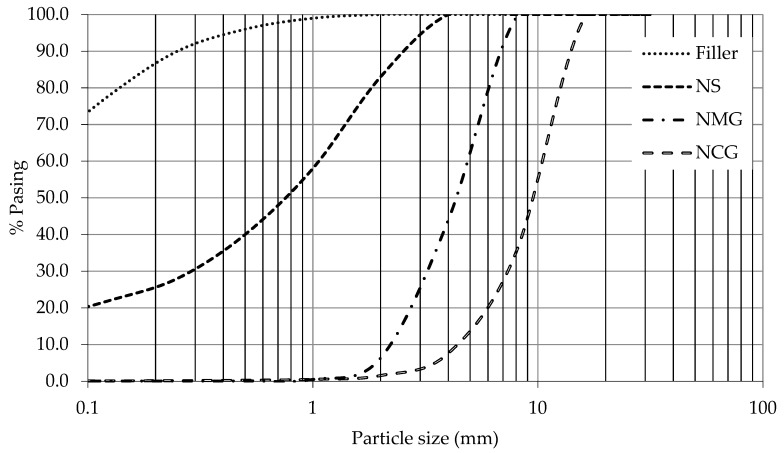
Particle size distribution curve.

The filler used is a crushed material of a limestone-dolomitic nature supplied by the company “Triturados Puerto Blanco” in Huétor-Santillán (Granada, Spain), and its density is 2.830 kg/m^3^. Its grain size is shown in Figure 1, and its specific surface has shown a value of 0.700 m^2^/g.

### 2.3. Natural Sand (NS)

The sand is of a limestone-dolomitic nature and is practically free of fines. It comes from the El Rayo quarry, Hermanos Guerrero, Loja (Granada, Spain).

### 2.4. Natural Coarse Gravel (NCG) and Natural Medium Gravel (NMG)

The aggregates used in the manufacture of concrete have a maximum size of 16 mm, the main properties are shown in Table 2 (Standards used in the experimental work are shown in Appendix A), and their granulometries are shown in Figure 1. The total gravel has been obtained by mixing 4/8 mm medium gravel (NMG) and 8/16 mm coarse gravel (NCG) from the Quintos quarry located in Huétor-Santillán (Granada).

### 2.5. Biomass Bottom Ash (BBA)

This is the coarse fraction produced in the primary combustion chamber due to incomplete combustion (decrease in the melting point) and is made up of most of the mineral fraction of the original biomass [30]. The BBA comes from the biomass power plant called Bioeléctrica de Linares S.L. from the company Sacyr Industrial located in the Linares-Baeza station, Jaén, Spain.

The fuel supply of the biomass used for the generation of electricity is made up of approximately 40% olive cake and 60% biomass of wood (olive, pine and eucalyptus). The BBAs were processed in the laboratory to obtain two different materials used in the manufacture of SCC: BBA (biomass bottom ash kiln-dried for 24 h and sieved with a particle size no larger than 5 mm) and BBA-C (biomass ash kiln-dried for 24 h and crushed, with a particle size no larger than 0.25 mm). The physical and chemical properties of BBA are presented in Table 2.

The physical and chemical characteristics of BBA depend on the types and the different burned biomass used as fuel, in addition to the technology used in the electricity generation plant by burning biomass [31,32].

An important factor in the physical properties of BBAs is their high absorption and low density (19.83% and 1.73 g/cm^3^, respectively). Both parameters are important in the design of mixtures, where the presence of water and the volume of the material are conditioning factors for its manufacture [33].

The friability ratio is an important property from an engineering point of view. A friable material is characterised by the ease of fragmentation of its particles [34]. The BBA has a high coefficient of friability (31.8%), but according to the technical specifications (EHE-08) a coefficient lower than 40% is recommended for the manufacture of concrete, so the BBAs under study are suitable for manufacturing concrete.

The organic matter content in the BBA has shown to be a consequence of the efficiency of the biomass combustion plant [35]; according to other authors, the BBA sample does not present a high percentage of organic content matter [31,32].

Calcium was the main constituent and accounted for 22.03% by weight (as oxide) of the ash mass. In addition to calcium, BBA is characterised by the relatively high content of potassium (14.06% by weight), the presence of which resulted from the initial content of nutrient ingredients in the olive wood trimmings, in accordance with previous studies [36].

**Figure 2:**
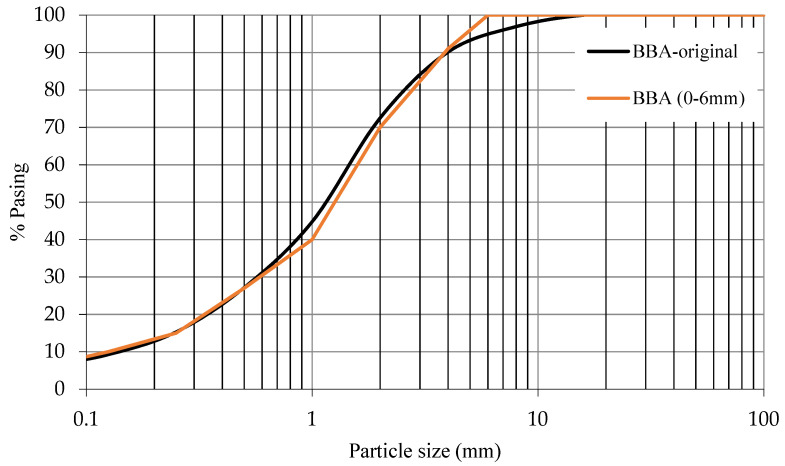
Particle size distribution curve of BBA original and BBA (0–6 mm).

**Figure 3:**
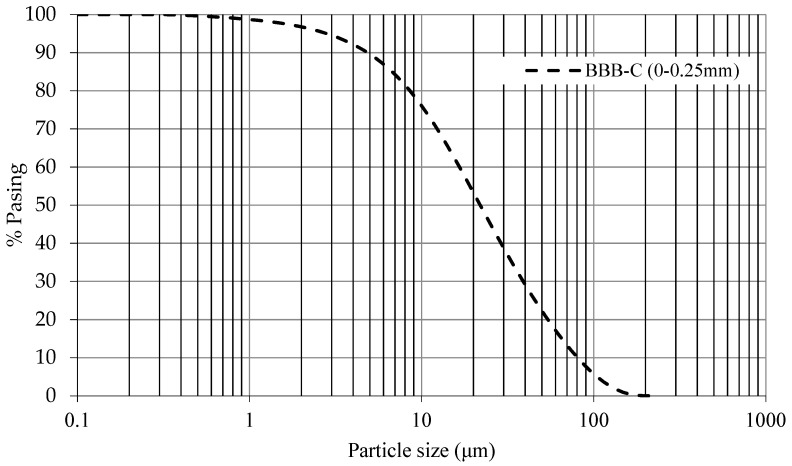
Particle size distribution curve of BBA-Crushed.

The particle size distribution of the BBA is shown in Figure 2 and Figure 3. Figure 2 shows the grain size curve of the BBA without any processing (BBA-original) and the BBA screened (BBA) to match the grain size of the sand. Figure 3 represents the grain size curve of the crushed BBA (BBA-C) achieving a grain size similar to that of the filler. All the particle size curves have a continuous grain size, thus, guaranteeing a lower percentage of voids in the manufacture of the concrete and optimising the necessary cement paste.

## 3. Dosage

The most important condition that must be considered in order to carry out the dosing of a self-compacting concrete is to provide a sufficient quantity of the set consisting of “cement + water + fines of less than 0.125 mm content in the aggregates” to achieve self-compacting characteristics.

Two series of dosages were carried out. The first series replaced filler with crushed biomass bottom ash (BBA-C) with a replacement of 20%, 40% and 60%, respectively, and the second series replaced natural sand with BBA (10%, 20% and 30%, respectively) without any processing. The additive used was MasterGlenium ACE 325 manufactured by BASF. Dosages are shown in Table 3.

### Workability of the Fresh Concrete

All the mixtures that underwent the tests indicated the current regulations (EHE-08) for the characterisation of self-compatibility: slump flow, according to EN 12350-8; V-funnel according to EN 12350-9; J-Ring according to EN 12350-12; and L-Box test according to EN 12350-10.

Slump flow: This is the simplest and most widely used method due to the simplicity of the equipment required. The value of the flow extension (Df) is useful for evaluating the deformation capacity of self-compacting concrete. Df measurements resulting between 60 and 80 cm are recommended, presenting mixtures in that range with good ability or ease for filling, according to EFNARC, 2002 [37].

J-Ring: This determines the ability of self-compacting concrete to flow through narrow openings, including gaps between trusses and other obstructions without segregation or blockage. After flow ceases, the final extension diameter is measured as the mean of two perpendicular diameters.

L-Box: This consists of a vertical tank that connects to a horizontal channel through an opening in which reinforcement bars are placed. The test involves filling the reservoir and allowing concrete to flow into the channel through the trusses. The time taken for the concrete to reach a distance of 200 mm (T20) and 400 mm (T40) is determined, and the heights H1 and H2 are reached at both ends of the horizontal part, with the mixture already at rest. The H2/H1 ratio is defined as the blocking coefficient (Cb).

V-funnel: This test evaluates the ability of concrete to flow in restricted areas in a vertical direction and under its own weight, qualifying the tendency with respect to segregation and blocking, by observing the variation in flow velocity.

**Figure 4:**
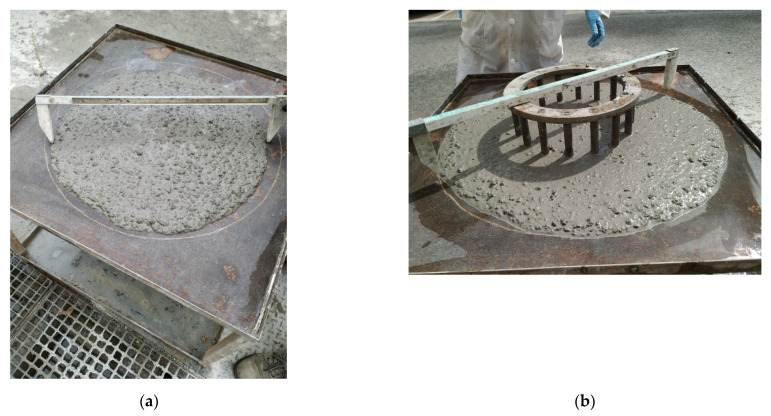
Results of the tests carried out according to the current regulations for considering self-compacting concrete (EHE-08). Slump flow (**a**) and J-Ring (**b**).

Table 4 shows the results of the tests carried out according to current regulations (EHE-08) for obtaining the condition of self-compacting. One can observe how all the dosages used are within the parameters imposed by the regulations, and Figure 4 shows the Slump flow test and the J-Ring.

## 4. Experimental Methods and Results

### 4.1. Compressive Strength of Test Specimens

The compressive strength was determined on 100 mm × 100 mm cubic samples for ages 7, 28, 90 and 256 days according to EN 12390-3: 2019. Table 5 shows the values of the resistances obtained for each of the series.

The use of industrial by-products as a substitute for the fine fraction directly affects simple compressive strength [38]. Some authors focus on the substitution of coarse and fine fraction of natural aggregates, obtaining substitution ranges depending on the type of industrial by-product used [39].

In this work, ranges of 20%, 40% and 60% substitution of limestone filler by BBA-C (Series 1) and 10%, 20% and 30% substitution of natural sand (Series 2) were proposed.

The best compressive strength results were shown for Series 1 where lime filler was replaced by BBA-C, which is essential to avoid SCC segregation.

**Figure 5:**
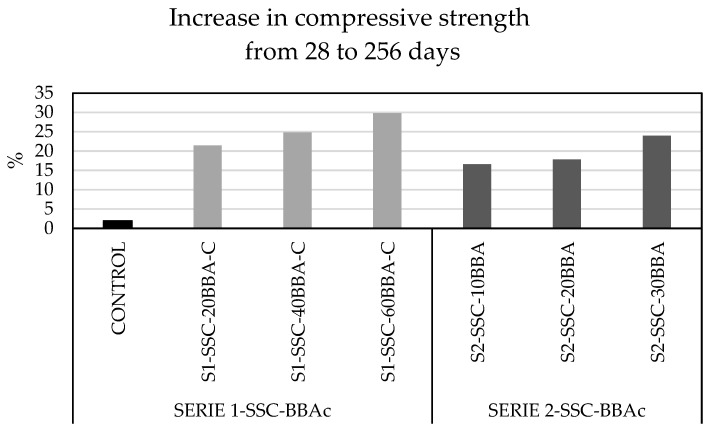
Comparative of increase in compressive strength from 28 to 256 days.

The analysis of the 28 day compressive strength showed a reduction in all mixes combined with biomass bottom ash, a result also observed by Mehta and Monteiro and Rosales et al. [40,41] However, Figure 5 shows the long-term increase in resistance. It can be observed that after 256 days, the mixes continue to gain strength approximately between 17% and 30%. This behaviour could be mainly attributed to the pozzolanicity of the BBA.

### 4.2. Tensile Splitting Strength of Test Specimens

This test was carried out by subjecting a 150 × 300 mm cylindrical specimen cured in a humid chamber for diametric compression for 28 days in accordance with EN 12390-6: 2009. A load was applied evenly along two opposite lines until breakage was achieved. This load causes a relatively uniform tensile stress throughout the diameter of the vertical load plane, and this tension is the one that exhausts the specimen and triggers the break in the diametral plane. The values obtained are shown in Table 6.

### 4.3. Determination of Modulus of Elasticity

The modulus of elasticity of concrete represents the stiffness of this material when faced with a load imposed on it. The test for the determination of the static modulus of elasticity of concrete is carried out by means of the EN 12390-13: 2014 Standard and has as its principle the application of static load and the corresponding produced unit deformation.

The first phase (elastic zone) applied a stress of 30% of the compressive strength obtained in the previously described test. In the second phase (curved line), the concrete specimen is submitted to breakage according to the regulations. The results are shown in Table 6.

Regarding the tensile splitting strength, the trend of the experimental data tends to guarantee the existing relationship between compression and traction in concrete. Tensile splitting strength values were observed in the concrete representing approximately 10–15% of the results obtained for compressive strength.

**Figure 6:**
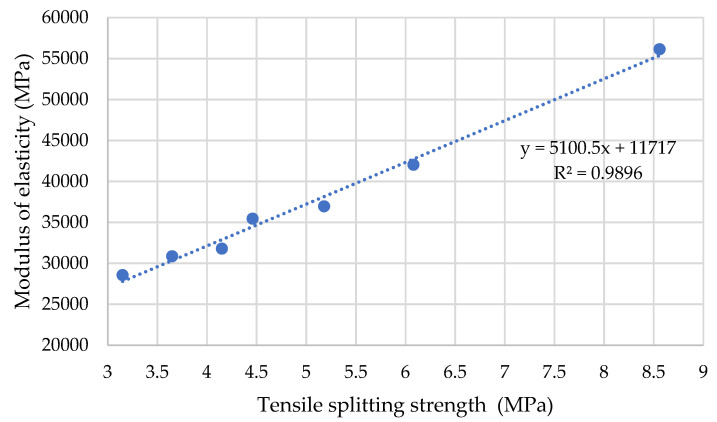
Relationship between tensile splitting strength and modulus of elasticity.

In order to observe the relationship between tensile splitting strength and the modulus of elasticity, a trend is proposed which aims to observe the behaviour of these two variables (Figure 6).

The modulus of elasticity ranged from 42.01 GPa to 31.77 GPa for Series 1 and between 35.43 GPa and 28.54 GPa for Series 2. These results demonstrate that the manufactured concrete can be suitable for many structural applications, even for structures with demanding limitations regarding serviceability limit states (e.g., deformations and deflections) [42].

### 4.4. Density and Absorption of Hardened Concrete

The density and absorption percentage were determined in specimens of hardened SCC. Each series was subjected to a cycle of immersion in water for 24 h and subsequently to oven drying between 105 °C and 110 °C for 24 h. With this procedure, the apparent mass in the water, the saturated mass with a dry surface and the dry mass are obtained, and the density and supply are obtained with these data in accordance with the EN 12390-7: 2019 standard (Testing hardened concrete—Part 7: Density of hardened concrete). The test results are shown in Table 7.

**Figure 7:**
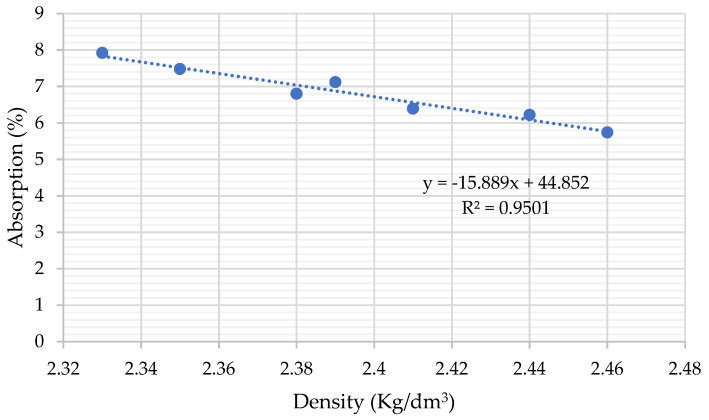
Relationship between density and absorption.

The results show how the incorporation of BBA decreases density and increases absorption in all the mixtures (Figure 7). Density directly depends on the density of the materials used to manufacture the concrete. In both series, the maximum BBA substitution corresponds to the lowest density and highest absorption values, and this is due to the fact that BBAs have a very porous structure in agreement with other authors [31,41].

### 4.5. Penetration of Water under Pressure

The depth of water penetration under pressure was determined in 150 × 300 mm cylindrical specimens cured in a humidity chamber for 28 days. The water was applied under pressure of 500 kPa for 72 h. Subsequently, the test piece was divided by breaking it into two halves, and the penetration of the waterfront was recorded in accordance with the EN 12390-8:2019 standard. The results are shown in Table 8.

Structural Concrete Instruction (EHE08) uses the determination of water penetration depth as a verifier that the concrete has sufficient impermeability to ensure durability during the service life of the structure.

The limits of the Structural Concrete Instruction are set at 50 mm for maximum depth and 30 mm for the average depth in mass or reinforced concrete.

As shown in Table 8, the differently manufactured concrete did not exceed the established limits. The higher water penetration values correspond to the higher BBA replacement rate, mainly due to the high porosity of the BBA [26].

### 4.6. Carbonatation Depth

Carbonation is a process of chemical origin that consists of the combination of CO_2_ with concrete portlandite. In order to know the degree of carbonation, the specimens were subjected to a CO_2_ saturated environment according to UNE 112011:2011 (relative humidity 55–65%, a temperature of 23 ± 3 °C and an air supply with 5 ± 0.1% CO_2_). To carry out the test, a piece of concrete was broken from each of the specimens, then the phenolphthalein solution was applied to the concrete. A colour change (pink) on the application surface will indicate that the concrete is not carbonated. If the colouration does not occur, it means that it is an area that is already carbonated.

**Figure 8:**
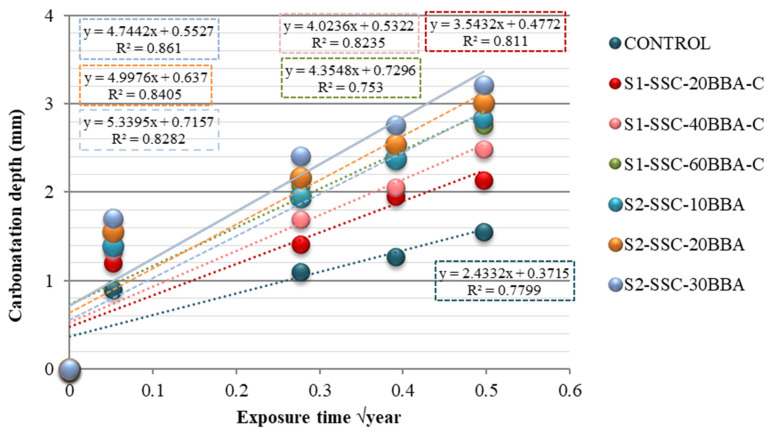
Relationship between carbonatation depth and exposure time.

From a chemical point of view, the carbonation of concrete produces a decrease in pH, which occurs when CO_2_ penetrates through the capillary pore network reacting with the moisture present and converting calcium hydroxide (high pH) to carbonates, which are neutral, reducing the alkalinity of the concrete. From a physical point of view, the carbonation of concrete depends directly on the porosity and permeability of the concrete, as well as the environmental conditions to which it is exposed [43]. Most carbonation models consider the amount of cement as an important parameter to be considered because of its strong influence on the reaction of CO_2_ with the Ca(OH)2 formed during cement hydration. Figure 8 shows the evolution over time of the carbonation of the concrete studied. According to the literature [44], the mixes with the highest porosity are also those with the highest carbonation, and the mix with a 30% substitution of BBA by sand (S2-SSC-30BBA) is the one that shows the highest carbonation front.

## 5. Conclusions

This study has evaluated the properties of biomass bottom ash with different treatments applied and the influence that its use has on the mechanical and durability properties in the manufacture of self-compacting concrete.

The following conclusions were obtained:

- An important factor in the physical properties of BBAs is their high absorption and low density (19.83% and 1.73 g/cm^3^, respectively). Both parameters are important in the design of mixtures where the presence of water and the volume of material is a conditioning factor for its manufacture.
- The presence of K_2_O in cementitious materials can reduce durability due to deterioration of the microstructure. However, the Si/Ca values of the mixture provide increased mechanical strength due to their pozzolanic character.
- In relation to the mechanical properties, compressive strength, tensile splitting strength and modulus of elasticity are reduced for all mixes combined with biomass bottom ash. However, the mixtures combined with biomass bottom ash still gain 17–30% in long-term compressive strength compared to the control mixtures.
- In terms of durability parameters, water penetration under pressure is higher when biomass bottom ash is incorporated with sand due to the larger particle size and high porosity of the mixture.
- The depth of carbonation of concrete depends on many variables, and the most important ones are porosity and permeability. Mixtures with higher porosity and permeability are also those with higher carbonation.

In conclusion, the use of self-compacting concretes with the substitution of up to 30% of natural sand by screened BBA and up to 60% of filler by crushed BBA is recommended for use in civil infrastructure works. The valorisation of biomass bottom ash instead of exploiting natural or non-renewable resources can eliminate the negative impact associated with indiscriminate disposal of this by-product in landfills.

## Figures and Tables

**Table 1:** Chemical properties of cement. CEM I 42.5-R.

Content (%)									
SiO_2_	Al_2_O_3_	FeO_3_	Cao	MgO	SO_3_	K_2_O	Na_2_O	Cl	Loss of Ignition
19.31	1.4	4.45	66.01	1.26	3.3	0.35	0.08	0.01	1.42

**Table 2:** Physical and chemical properties of natural aggregates and BBA.

PROPERTIES		NS	NCG	NMG	BBA	Test Method
Density-SSD (kg/m^3^)		2.53	2.65	2.62	1.73	EN 1097-6: 2014
Water absorption (%)		0.9	1.59	1.63	19.83	EN 1097-6:2014
Los Angeles (%)		-	17	18		
Friability ratio (%)		-			31.8	146404: 2018
Sand equivalent (%)		85	-	-	23	EN 933-8:2012
Plasticity		Non plastic	Non plastic	Non plastic	Non plastic	EN ISO 17892-12:2019
Chlorides					0.15	EN 1774-1:2010
Organic matter %					1.51	UNE 103204:2019
Water-soluble sulphate (%SO_4_)		<0.01	<0.01	<0.01	0.32	EN 1744-1:2010
Acid-soluble sulphate (%SO_4_)		<0.01	<0.01	<0.01	0.33	EN 1744-1:2010
Elemental content (%)						EN 196-2:2014
SiO_2_		-	-	-	37.67	
CaO		-	-	-	22.03	
K_2_O		-	-	-	14.06	
MgO		-	-	-	5.95	
Fe_2_O_3_		-	-	-	2.52	
Al_2_O_3_		-	-	-	3.02	

**Table 3:** Concrete mix proportions (kg/m^3^).

Serie	Mix Name	Natural Aggregates				Biomass Bottom Ash		CEM	Water	W/C Relation	Additive
		NCG	NMG	NS	Filler	BBA-C	BBA				
	CONTROL	487.62	151.1	989.3	185	-	-	400	200	0.5	3.6
SERIE 1-SSC-BBAc	S1-SSC-20BBA-C	487.62	151.1	989.3	148	37	-		200	0.5	4.8
	S1-SSC-40BBA-C	487.62	151.1	989.3	111	74	-		200	0.5	8.0
	S1-SSC-60BBA-C	487.62	151.1	989.3	74	111	-		200	0.5	8.4
SERIE 2-SSC-BBA	S2-SSC-10BBA	487.62	151.1	890.67	185	-	98.63	400	200	0.5	4.8
	S2-SSC-20BBA	487.62	151.1	791.44	185	-	197.86		200	0.5	6.8
	S2-SSC-30BBA	487.62	151.1	692.51	185	-	296.79		200	0.5	7.0

**Table 4:** Results of the tests carried out according to the current regulations for considering self-compacting concrete (EHE-08).

	Test	Measured Parameter	Test Result	Permissible Range (EHE-08)
SSC-Control	Slump flow	T_50_ (s)	2.8	
		d_f_ (mm)	690	
	J-Ring	dj_f_ (mm)	650	
	L-Box	Cb	0.78	
	V-funnel	Tv (s)	5.02	
SSC-20BBA-C	Slump flow	T_50_ (s)	1.5	
		df (mm)	650	
	J-Ring	djf (mm)	650	
	L-Box	Cb	0.75	
	V-funnel	Tv (s)	4.6	
SSC-40BBA-C	Slump flow	T_50_ (s)	1.6	
		df (mm)	660	
	J-Ring	djf (mm)	670	
	L-Box	Cb	0.78	
	V-funnel	Tv (s)	5.9	
SSC-60BBA-C	Slump flow	T_50_ (s)	2.1	T50 ≤ 8 s (s)
		df (mm)	760	550 mm ≤ df ≤ 850 mm
	J-Ring	djf (mm)	740	≥df-50 mm
	L-Box	Cb	0.75	0.75 ≤ Cb ≤ 1.00
	V-funnel	Tv (s)	7.9	4 s ≤ Tv ≤ 20 s
SSC-10BBA	Slump flow	T_50_ (s)	3.6	
		df (mm)	660	
	J-Ring	djf (mm)	610	
	L-Box	Cb	0.79	
	V-funnel	Tv (s)	10	
SSC-20BBA	Slump flow	T50 (s)	4	
		df (mm)	690	
	J-Ring	djf (mm)	650	
	L-Box	CbL	0.8	
	V-funnel	Tv (s)	10	
SSC-30BBA	Slump flow	T50 (s)	4.3	
		df (mm)	640	
	J-Ring	djf (mm)	630	
	L-Box	CbL	0.81	
	V-funnel	Tv (s)	11	

**Table 5:** Compressive strength of SCC.

		Compressive Strength (MPa)				
Time (Days)		7	28	90	256	INCREASING28–256 DAYS (%)
	CONTROL	67.26	81.53	82.87	83.14	1.90
SERIE 1-SSC-BBA-C	S1-SSC-20BBA-C	53.06	59.62	65.76	72.43	21.48
	S1-SSC-40BBA-C	43.11	51.93	56.69	64.81	24.80
	S1-SSC-60BBA-C	38.74	45.21	49.12	58.67	29.77
SERIE 2-SSC-BBA	S2-SSC-10BBA	43.29	50.12	53.28	58.44	16.60
	S2-SSC-20BBA	37.76	43.51	47.96	51.27	17.83
	S2-SSC-30BBA	35.58	39.48	43.55	48.94	23.96

**Table 6:** Tensile splitting strength and modulus of elasticity.

		SERIE 1-SSC-BBA-C			SERIE 2-SSC-BBA		
	CONTROL	S1-SSC-20BBA-C	S1-SSC-40BBA-C	S1-SSC-60BBA-C	S2-SSC-10BBA	S2-SSC-20BBA	S2-SSC-30BBA
Tensile splitting strength (MPa)	8.56	6.08	5.18	4.15	4.46	3.65	3.15
Modulus of elasticity (MPa)	56,129	42,018	36,947	31,779	35,439	30,857	28,541

**Table 7:** Density and absorption of hardened concrete.

		SERIE 1-SSC-BBA-C			SERIE 2-SSC-BBA		
	CONTROL	S1-SSC-20BBA-C	S1-SSC-40BBA-C	S1-SSC-60BBA-C	S2-SSC-10BBA	S2-SSC-20BBA	S2-SSC-30BBA
Density (Kg/dm^3^)	2.46	2.44	2.41	2.38	2.39	2.35	2.33
Absorption (%)	5.74	6.22	6.39	6.8	7.12	7.48	7.92

**Table 8:** Penetration of water under pressure (mm).

		SERIE 1-SSC-BBA-C			SERIE 2-SSC-BBA		
	CONTROL	S1-SSC-20BBA-C	S1-SSC-40BBA-C	S1-SSC-60BBA-C	S2-SSC-10BBA	S2-SSC-20BBA	S2-SSC-30BBA
Penetration of water under pressure (mm)	3.12	5.37	6.89	7.54	6.57	8.15	9.87

## Appendix A

Standards used in the experimental work are as follows:

EN 1097-6:2014. Tests for mechanical and physical properties of aggregates—Part 6: Determination of particle density and water absorption.

EN 146404: 2018. Aggregates for concrete determination of the coefficient of friability of the sands.

EN 933-8:2012. Tests for geometrical properties of aggregates—Part 8: Assessment of fines—Sand equivalent test.

EN ISO 17892-12:2019. Geotechnical investigation and testing—Laboratory testing of soil—Part 12: Determination of liquid and plastic limits (ISO 17892-12:2018).

EN 1744-1:2010. Tests for chemical properties of aggregates—Part 1: Chemical analysis.

UNE 103204:2019. Organic matter content of a soil by the potassium permanganate method.

UNE -EN 196-2:2014. Method of testing cement—Part 2: Chemical analysis of cement.

Real Decreto 1247/2008, de 18 de julio, por el que se aprueba la instrucción de hormigón estructural (EHE-08), Boletín Oficial del Estado, núm. 203, de 22 de agosto de 2008, pp. 35176 a 35178.

UNE-EN 12350-8:2011. Testing fresh concrete—Part 8: Self-compacting concrete—Slump-flow test.

UNE-EN 12350-9:2011. Testing fresh concrete—Part 9: Self-compacting concrete—V-funnel test.

UNE-EN 12350-10:2011. Testing fresh concrete—Part 10: Self-compacting concrete—L box test.

UNE 12350-12:2011. Testing fresh concrete—Part 12: Self-compacting concrete—J-ring test.

UNE 12390-3:2019. Testing hardened concrete—Part 3: Compressive strength of test specimens.

UNE 12390-6:2010. Testing hardened concrete—Part 6: Tensile splitting strength of test specimens.

EN 12390-13:2014. Testing hardened concrete—Part 13: Determination of secant modulus of elasticity in compression.

EN 12390-7:2019. Testing hardened concrete—Part 7: Density of hardened concrete.

EN 12390-8:2019. Testing hardened concrete—Part 8: Depth of penetration of water under pressure.

UNE 112011:2011. Corrosion of concrete reinforcement steel. Determination of the carbonatation depth for in-service concrete.
